# Feasibility of ultrahigh field (7 Tesla) human cardiovascular magnetic resonance imaging to assess cardiac volumes and mass validated against 1.5 T and 3T field strengths

**DOI:** 10.1186/1532-429X-13-S1-O45

**Published:** 2011-02-02

**Authors:** Joseph J Suttie, Lance DeLaBarre, Alex Pitcher, Pierre-Francois van de Moortele, Sairia Dass, Carl J Snyder, Jane M Francis, Greg J Metzger, Peter Weale, Kamil Ugurbil, Matthew D Robson, Stefan Neubauer, Tommy Vaughan

**Affiliations:** 1 Unversity of Oxford, Oxford, UK; 2 Unversity of Minnesota, Minneapolis, MN, USA; 3 Siemens Healthcare, Chicago, IL, USA; 4 University of Minnesota, Minneapolis, MN, USA

## Introduction

Ultrahigh (7T) cardiovascular magnetic resonance imaging (CMR) is an emerging field of clinical research because theoretically higher signal to noise offers potential benefits for imaging coronaries, perfusion and spectroscopy. We report the first comparison of CMR at 1.5 T, 3 T and 7 T field strengths using steady state free precession (SSFP) and fast low angle shot (FLASH) cine sequences.

## Methods

**Figure 1:**
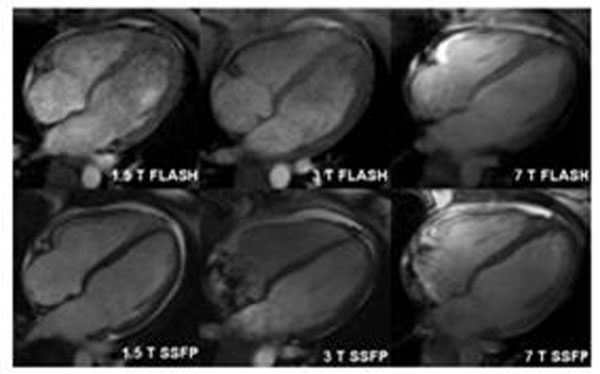
Horizontal long axis (HLA) cardiac images acquired at 1.5, 3 and 7 T using fast low angle shot (FLASH) and steady state free precession (SSFP) sequences in the same subject.

Ten volunteers underwent retrospectively ECG gated CMR at 1.5 T, 3 T and 7 T using FLASH and SSFP sequences (Figure 1). B1 and B0 shimming, and frequency scouts were used to optimise image quality.

## Results

**Figure 2:**
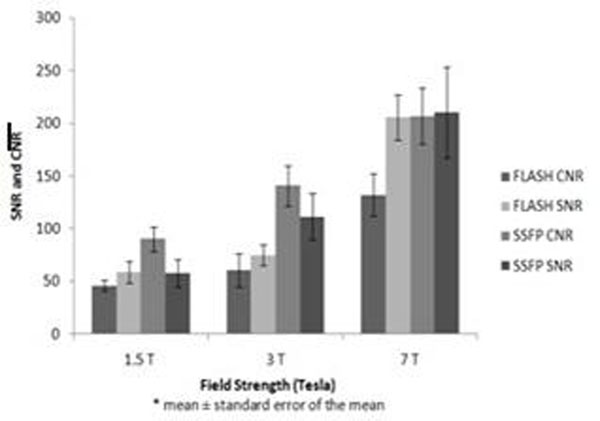
Myocardial signal to noise (SNR) and blood to myocardial contrast to noise ratios (CNR) for SSFP and FLASH sequences at 1.5 Tesla (T), 3 T and 7 T field strengths.

Cardiac volume and mass measurements were not significantly affected by field strength when using the same imaging sequence (P>0.05 for all parameters at 1.5 T, 3 T and 7 T). SSFP imaging returned larger end diastolic and end systolic volumes and smaller left ventricular masses than FLASH imaging at 7 T and at the lower field strengths (P<0.05 for each parameter). There was a smaller difference between volumes and mass measurements between SSFP and FLASH imaging at 7 T than 1.5 T and 3 T. Signal to noise (SNR) and contrast to noise (CNR) ratios were significantly higher at 7 T than the lower field strengths for both both SSFP (SNR 1.5 T:58, 3 T:112, 7 T:211; CNR 1.5 T:91, 3 T:141, 7 T: 208) and FLASH (SNR 1.5 T:59, 3 T:75, 7 T:206; CNR 1.5 T:46, 3 T:61; 7 T:133) (P<0.05 all comparisons)(Figure 2).

## Conclusions

SSFP and FLASH cine imaging at 7 T is technically feasible and provides valid assessment of cardiac volumes and mass compared with CMR imaging at 1.5 T and 3 T field strengths.

